# A fluorescence imaging technique suggests that sweat leakage in the epidermis contributes to the pathomechanism of palmoplantar pustulosis

**DOI:** 10.1038/s41598-023-50875-x

**Published:** 2024-01-03

**Authors:** Kazuki Yatsuzuka, Ryosuke Kawakami, Yosuke Niko, Teruko Tsuda, Kenji Kameda, Nobushige Kohri, Satoshi Yoshida, Ken Shiraishi, Jun Muto, Hideki Mori, Yasuhiro Fujisawa, Takeshi Imamura, Masamoto Murakami

**Affiliations:** 1 Department of Dermatology, Ehime University Graduate School of Medicine, Ehime, Japan; 2 Department of Molecular Medicine for Pathogenesis, Ehime University Graduate School of Medicine, Ehime, Japan; 3 Research and Education Faculty, Multidisciplinary Science Cluster, Interdisciplinary Science Unit, Kochi University, Kochi, Japan

**Keywords:** Skin diseases, 3-D reconstruction, Fluorescence imaging, Time-lapse imaging, Anatomy

## Abstract

Sweat is an essential protection system for the body, but its failure can result in pathologic conditions, including several skin diseases, such as palmoplantar pustulosis (PPP). As reduced intraepidermal E-cadherin expression in skin lesions was confirmed in PPP skin lesions, a role for interleukin (IL)-1-rich sweat in PPP has been proposed, and IL-1 has been implicated in the altered E-cadherin expression observed in both cultured keratinocytes and mice epidermis. For further investigation, live imaging of sweat perspiration on a mouse toe-pad under two-photon excitation microscopy was performed using a novel fluorescent dye cocktail (which we named JSAC). Finally, intraepidermal vesicle formation which is the main cause of PPP pathogenesis was successfully induced using our "LASER-snipe" technique with JSAC. "LASER-snipe" is a type of laser ablation technique that uses two-photon absorption of fluorescent material to destroy a few acrosyringium cells at a pinpoint location in three-dimensional space of living tissue to cause eccrine sweat leakage. These observatory techniques and this mouse model may be useful not only in live imaging for physiological phenomena in vivo such as PPP pathomechanism investigation, but also for the field of functional physiological morphology.

## Introduction

Sweat is the body’s surface thermal control system but in human skin it plays other roles as well, acting as a moisturizer, an inactivator of allergens, and in the protection against infection based on the secretion of antimicrobial peptides^1,2^. Given these multiple functions of sweat, its failure results in the development of several diseases. While common skin diseases such as miliaria and eczema can be treated with topical agents, palmoplantar pustulosis (PPP), a chronic inflammatory skin disease, is often resistant to treatment. In Japan, PPP has a prevalence of 0.12%^3^.

The main clinical manifestations of PPP are erythema, vesicles, pustulo-vesicles, pustules, and scales on the palms and soles. Typical lesions are usually localized, but may affect the patient's quality of life^4^; in severe cases patients may have difficulty walking due to the intense pain.

Based on its skin manifestation, PPP is regarded as a subtype of psoriasis referred to as pustular psoriasis but it has also been argued that it is a distinct disease. Pustular psoriasis is often classified into localized and generalized form^5^. Enhanced IL-36 signaling is a crucial pathway in generalized pustular psoriasis^6,7^. On the other hand, PPP, one of the localized pustular psoriasis, is sometimes associated with plaque psoriasis, but it is proposed not to formally include this under the heading "psoriasis" in its clinical, epidemiological, genetic, and biological features^5^. Its pathogenesis is unclear but two types of PPP, type-A and type-B, have long been recognized^4^. Most Japanese PPP patients have the type-A form. Histopathologically, type-A PPP skin lesions typically begin with vesicle formation, followed in turn by pustulo-vesicles and, finally, pustules^4^. The pustulo-vesicle and pustule phases have been well studied but little is known about the vesicle phase, and it may provide insights into the pathomechanism of PPP.

The most common site of PPP development is around the acrosyringium. The lesion begins as a small vesicle^4,8^ containing the eccrine sweat-derived antimicrobial peptides dermcidin and hCAP-18/LL-37^8,9^. These findings strongly suggest a relationship between eccrine sweat and intraepidermal vesicle formation in PPP. We have already succeeded in showing that the direct evidence for this relationship was obtained using our three-dimensional, deep-imaging technique with a new pyrene probe and two-photon excitation microscopy (TPM)^10^. In contrast to routine histopathology with hematoxylin–eosin (H&E) staining, this approach can reveal the three-dimensional structure of the acrosyringium.

In the development of eczema, spongiosis formation is accompanied by hyaluronan production and decreased E-cadherin expression by cytokine-stimulated keratinocytes^11^. In pompholyx, a type of vesicular dermatitis, small vesicles are seen in the lesion epidermis, and the clinical and histopathological features resemble those of PPP^12,13^. We previously showed that keratinocytes around the vesicles or pustules of PPP do not express hyaluronan, nor is hyaluronan present in the intercellular space of the epidermal keratinocytes in pompholyx^14^. Those observations suggest that the mechanism of vesicle formation in PPP differs from that in eczema.

In human sweat from the palms and soles, interleukin (IL)-1α and IL-1β concentrations are higher than in sweat from other parts of the body^15,16^. IL-1β decreases E-cadherin expression by primary normal human epidermal keratinocytes (NHEKs), leading to almost complete cell dissociation^17^. Neither IL-1α nor IL-1β increases hyaluronan production by primary NHEKs^11,18^. Based on these findings, we hypothesized that, in PPP, IL-1-rich acral eccrine sweat leaking in the epidermis leads to intraepidermal vesicle formation without an eczematous reaction.

To test this hypothesis, we developed a high-quality fluorescence imaging technique using a novel fluorescent dye cocktail (which we named JSAC). By combining our "LASER-snipe" technique with TPM, live imaging of sweat perspiration and real-time vesicle formation in the epidermis was possible in a mouse model of PPP.

## Results

### E-cadherin expression is decreased in the lesional epidermis of patients with PPP

**Figure 1:**
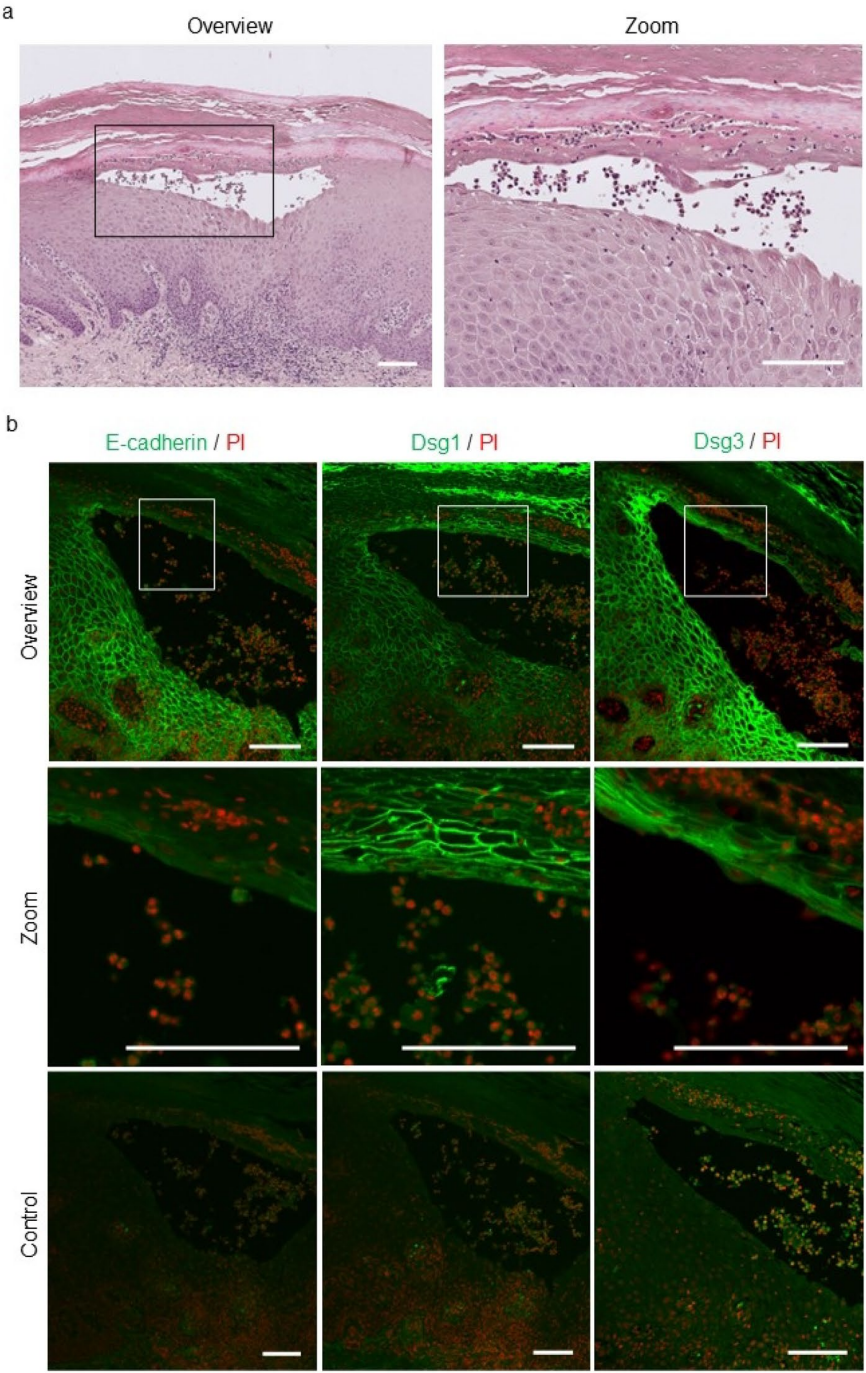
E-cadherin expression is decreased in the lesional epidermis of patients with PPP. (**a**) Representative image of H&E staining in human palmoplantar pustulosis (PPP) lesional skin (n = 3). Magnified areas are marked with black rectangles. Scale bar = 100 µm. (**b**) Representative images of immunofluorescent staining for E-cadherin (green), desmoglein-1 (green), and desmoglein-3 (green) in human PPP lesional skin (n = 3). Magnified areas are marked with white rectangles. The immunoglobulin fraction from non-immunized rabbits served as a negative control. Scale bar = 100 µm. PI, propidium iodide (red).

To explore the pathomechanism of intraepidermal vesiculation in PPP, lesional skin samples were obtained from three patients with PPP. Tissue sections prepared from samples were subjected to H&E (Fig. 1a) and immunofluorescence (Fig. 1b) staining to investigate the expression patterns of intercellular adhesional molecules (E-cadherin, desmoglein-1, and desmoglein-3) on epidermal keratinocytes. In normal human skin, classical and desmosomal cadherins are differentially expressed in different layers of the epidermis. E-cadherin is present in almost all layers while desmoglein-1 is dominant in upper layers and desmoglein-3 in lower layers^19,20^. Our focus was on the fluorescence signal intensity of Alexa-488-labeled E-cadherin and desmoglein-1 in the upper layers of the pustulo-vesicle (Fig. 1b, boxed area), which showed reduced E-cadherin fluorescence signal intensity compared to normal skin whereas there was no change in the fluorescence signal intensity of desmoglein-1 (Fig. 1b, zoom). The immunoglobulin fraction from non-immunized rabbits was used as a negative control to assess background signals; complete negative staining was routinely achieved using our standard protocol (Fig. 1b, control). We therefore hypothesized that decreased E-cadherin expression in the epidermis is associated with intraepidermal PPP vesicle formation.

### IL-1 diminishes E-cadherin expression in primary NHEKs

**Figure 2:**
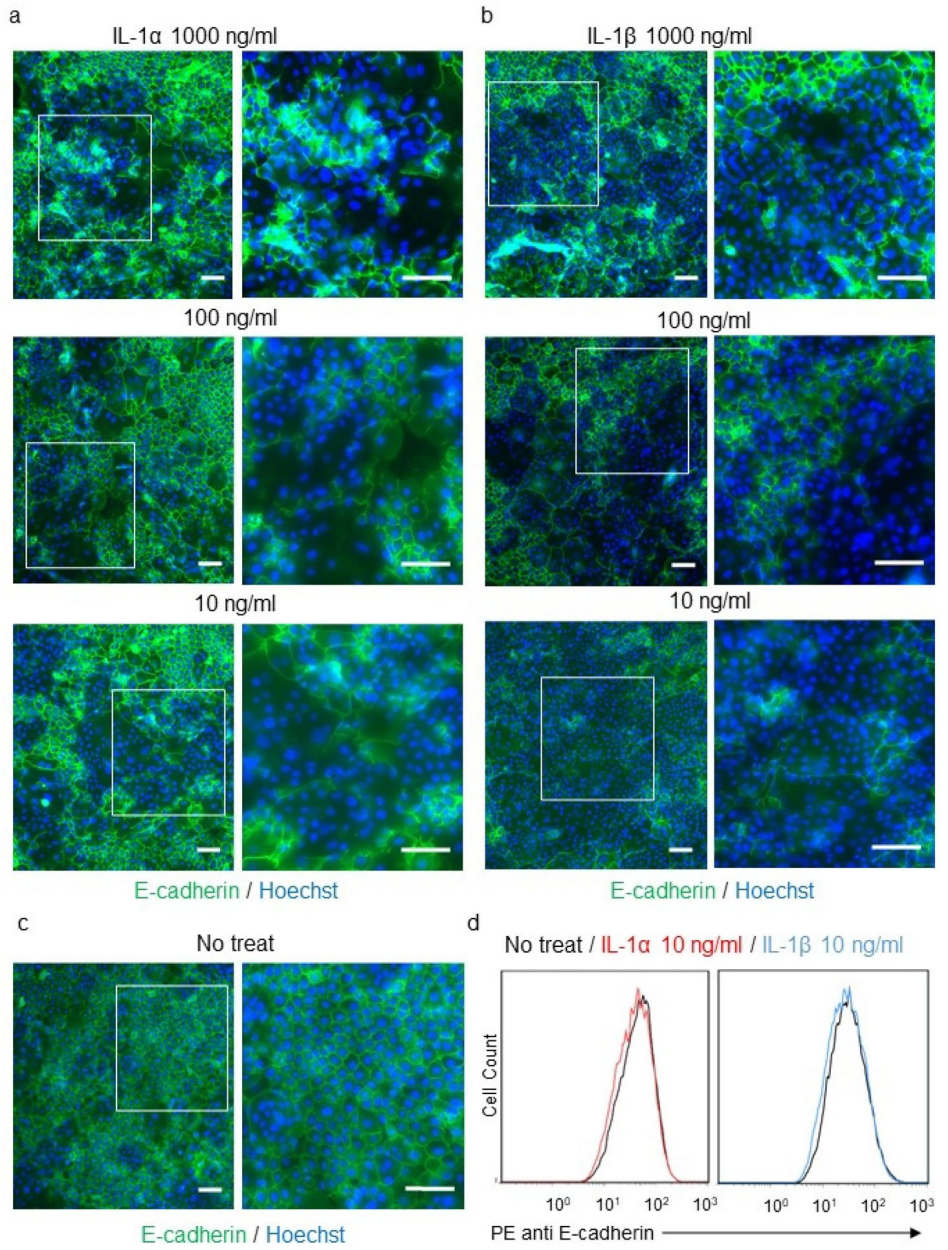
Interleukin (IL)-1 diminishes E-cadherin expression on the cell membrane of primary NHEKs. (**a**,**b**) Immunofluorescent microscopy images of NHEKs stimulated with IL-1α (**a**) or IL-1β (**b**) and stained with anti-E-cadherin (green) and Hoechst dye (blue). Magnified areas are marked with white rectangles. Scale bar = 100 µm. (**c**) Immunofluorescence microscopy images of non-treated NHEKs stained with anti-E-cadherin (green) and Hoechst dye (blue). Magnified areas are marked with white rectangles. Scale bar = 100 µm. (**d**) Representative data of E-cadherin expression in IL-1α- or IL-1β-treated NHEKs as determined by flow cytometry.

To determine whether IL-1, a cytokine present at high levels in acral human sweat, decreases epidermal E-cadherin expression, primary NHEKs were stimulated for 18 h with IL-1α and IL-1β at concentrations of 10, 100, or 1000 ng/ml (Fig. 2a–c). The results showed a decrease in fluorescence signal intensity in IL-1α (Fig. 2a) and IL-1β (Fig. 2b)-treated cells compared to the non-treated cells (Fig. 2c) at all concentrations. However, the action of IL-1 was not uniform, as a mosaic pattern area of normal E-cadherin expression was seen (Fig. 2a,b). A comparison of the parallel H&E-stained cultures showed no marked morphological difference between non-treated and treated cells (Supplementary Fig. 1). To further explore the lack of uniform IL-1 expression, the fluorescently stained primary NHEKs were stimulated for 18 h with 10 ng/ml IL-1α or IL-1β, after which E-cadherin expression was analyzed by multicolor flow cytometry. In line with the result of the immunocytochemical assay, E-cadherin expression was reduced in treated vs. non-treated cells (Fig. 2d) but there was no statistically significant difference in the mean fluorescence intensity (data not shown). These results suggested that IL-1α and IL-1β decrease the cell membrane expression of E-cadherin. Based on these results and those of previous studies showing an association between eccrine sweat, IL-1, acrosyringium structure, and PPP, we hypothesized that decreased E-cadherin expression, induced by IL-1-rich eccrine sweat from the palms and soles leaking into the epidermis, is the cause of intraepidermal vesicle formation in PPP. This hypothesis was tested in a mouse model, as described below.

### Three-dimensional, deep-imaging technique with TPM reveals numerous eccrine sweat glands in mouse toes and in humans

**Figure 3:**
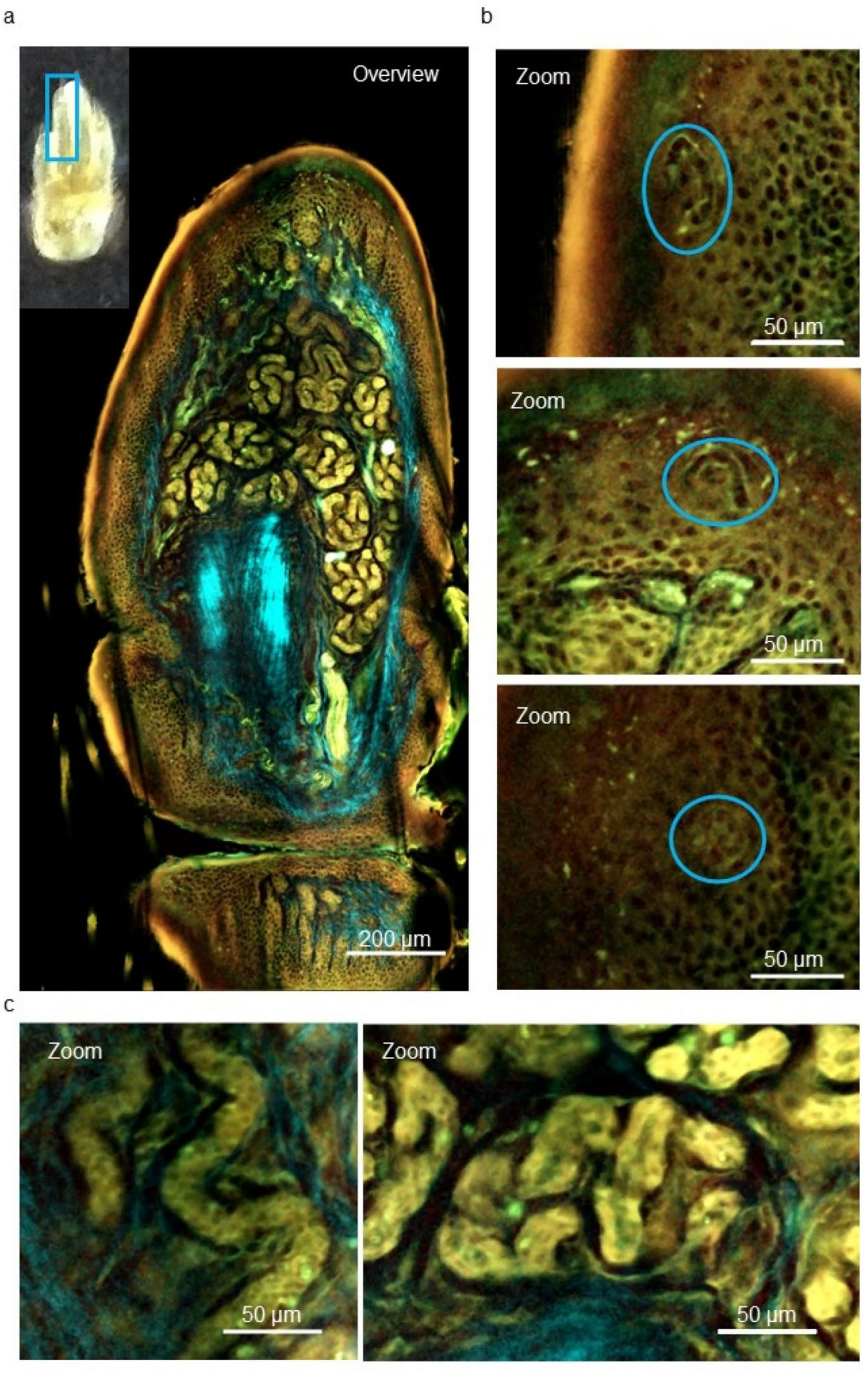
Three-dimensional and deep-imaging with two-photon excitation microscopy (TPM) reveals eccrine sweat glands in the mouse toe. (**a**) TPM image of the cleared mouse toe (marked with a blue rectangle) stained with a fluorescent solvatochromic pyrene probe (green). Scale bar = 200 µm. (**b**) Magnified images of the acrosyringium (marked with blue circles) in the epidermis of the mouse shown in (**a**). Scale bar = 50 µm. (**c**) Magnified images of the eccrine sweat ducts (left) and glands (right) in the dermis of the mouse shown in (**a**). Scale bar = 50 µm.

A recently established fluorescent solvatochromic pyrene probe was used to perform TPM for three-dimensional, deep imaging of a lipid-stained tissue sample from a C57BL/6 mouse toe. This technique clearly showed that, like the human toe, the mouse toe has numerous eccrine sweat glands (Fig. 3a, c and Supplementary Movie. 1). The acrosyringium and epidermal keratinocytes in the mouse toe were also visualized using this technique (Fig. 3b). These findings confirmed the validity of a mouse model for studies of sweat and the sweat ducts in skin disorders such as PPP as well as the use of a solvatochromic pyrene probe and TPM to study the underlying pathology.

### IL-1 diminishes E-cadherin expression by the epidermal keratinocytes of C57BL/6 mouse toe skin

**Figure 4:**
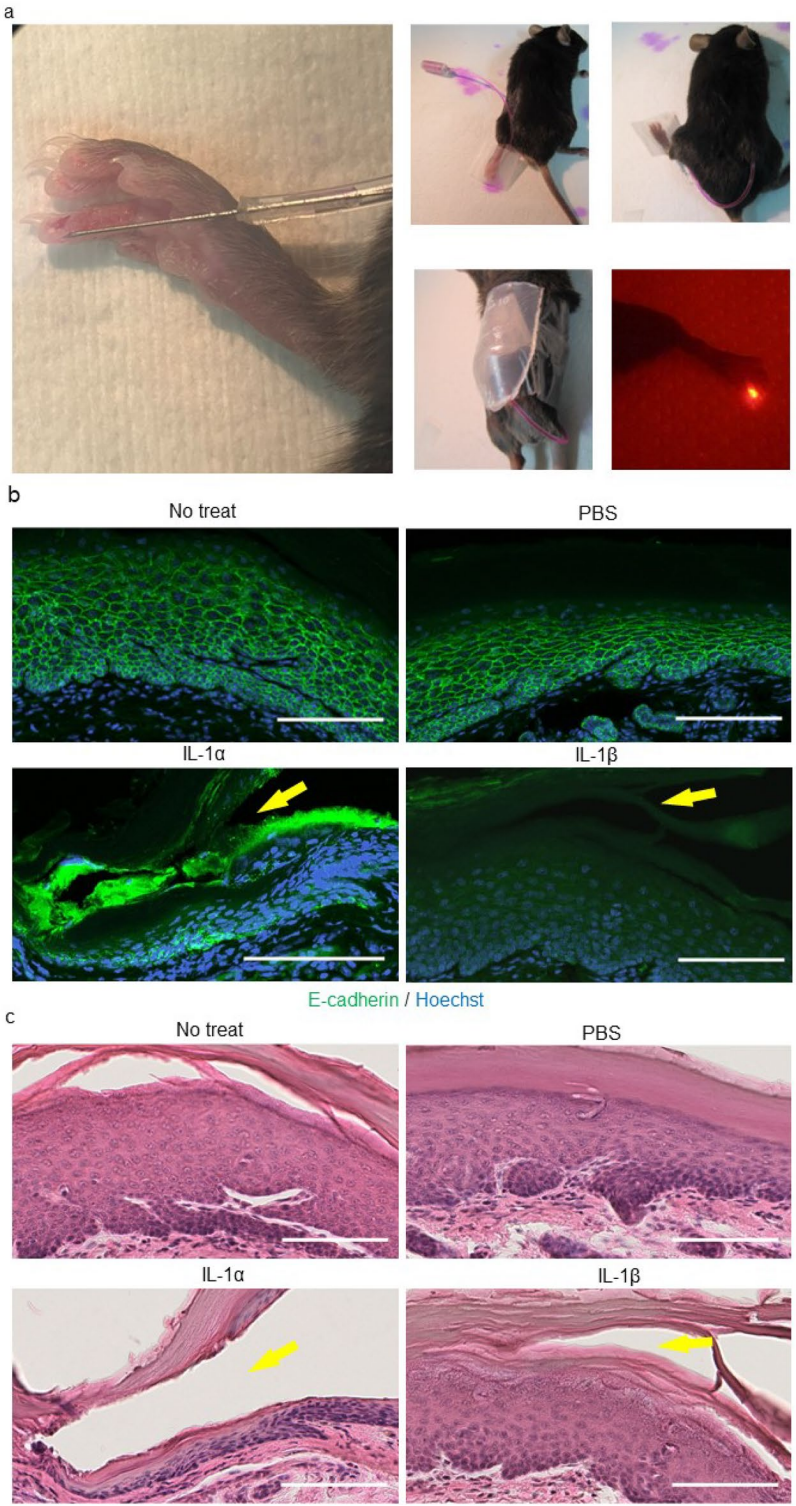
IL-1 diminishes E-cadherin expression on the cell membrane of epidermal keratinocytes in mouse toe. (**a**) Graphical depiction of the Alzet^®^ osmotic pump and cannula implantation in C57BL/6 mice under general anesthesia. Following administration of a continuous intraepidermal dose of 10 ng IL-1/ml mixed with SR101 to the mouse toe, a red fluorescence signal was detected (right, bottom). (**b**) Immunofluorescent microscopy images of mouse toe epidermis stimulated with PBS, IL-1α, and IL-1β and stained with anti-E-cadherin (green) and Hoechst dye (blue). (**c**) H&E staining of the specimens in (**b**). In (**b**) and (**c**), the yellow arrows indicate the needle insertion sites. Scale bars in (**b**) and (**c**) = 100 µm.

To demonstrate the effect of IL-1 on mouse epidermal keratinocytes, the subcorneal to spinous layers of mouse toe epidermis were stimulated for 24 h with 10 ng/ml IL-1α or IL-1β using an Alzet^®^ osmotic pump drug delivery system (Fig. 4a). In line with the immunocytochemistry results obtained in NHEKs (Fig. 2a–c), the fluorescence signal intensity of Alexa-488-labeled E-cadherin on the cell membranes of the treated epidermis was decreased compared to non-treated or phosphate-buffered saline (PBS)-treated epidermis (Fig. 4b). Again, there was no apparent difference in the epidermis of the respective H&E-stained specimens (Fig. 4c). These results suggested that, as in NHEKs, E-cadherin expression on the keratinocytes of mouse epidermis was diminished by IL-1α or IL-1β.

### Three-dimensional, deep-imaging with TPM together with a novel dye cocktail (JSAC) in the live imaging of perspiration on the mouse foot-pad

**Figure 5:**
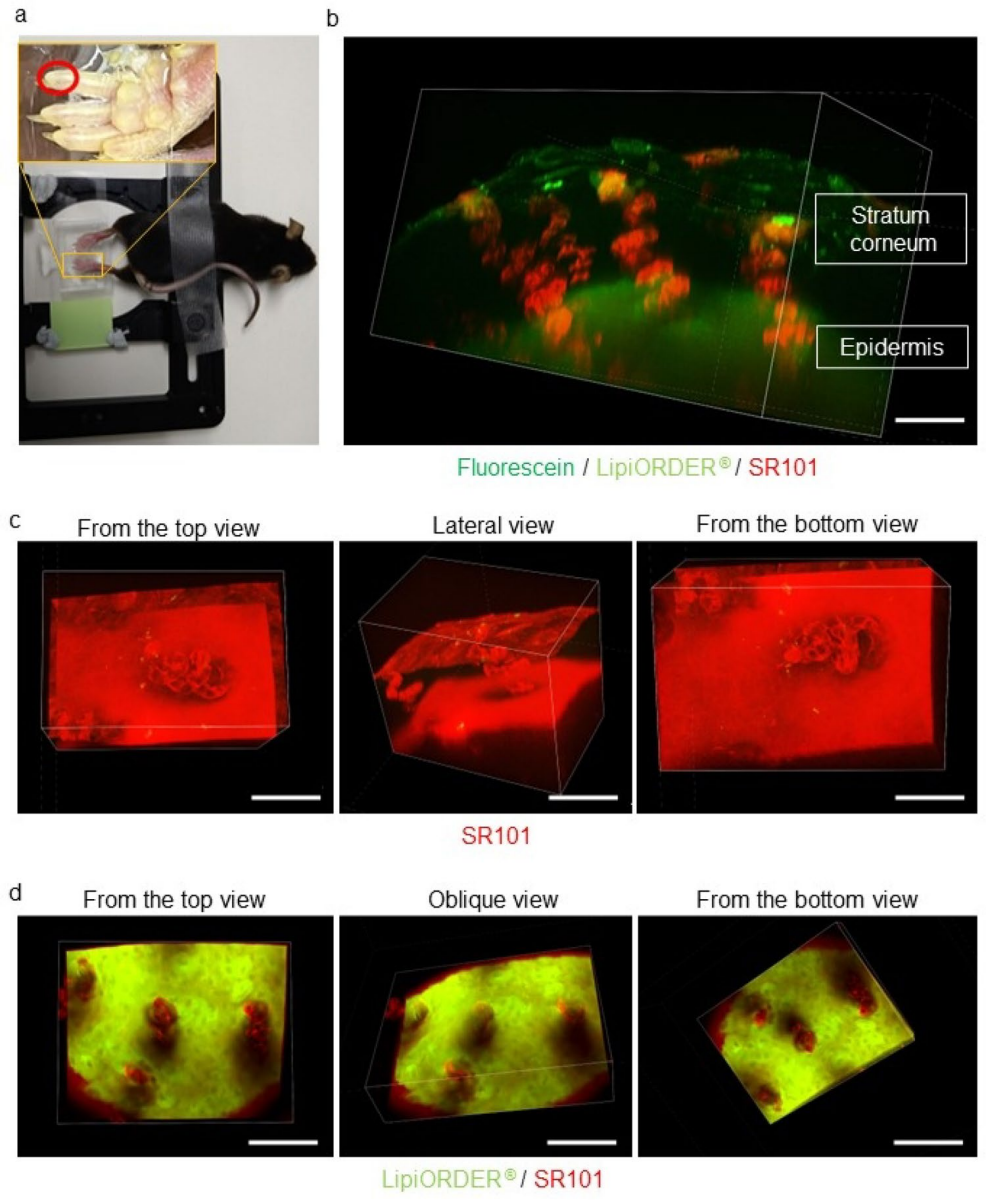
Three-dimensional, deep-imaging with TPM using a novel mixture of dyes in live imaging of perspiration on a mouse toe-pad. (**a**) Mice under general anesthesia were restrained for TPM live imaging. The observation field is indicated by a red circle. (**b**) Three-dimensional TPM image of the moment of perspiration on a mouse toe stained with a mixture of three dyes: fluorescent solvatochromic pyrene probe (light green), fluorescein (green), and SR101 (red). Scale bar = 100 µm. (**c**) Stratum-corneum-weighted image of (**a**) in top, lateral, and bottom views. Scale bar = 100 µm. (**d**) Epidermis-weighted image (**a**) viewed from the top, oblique, and bottom. Scale bar = 100 µm.

Three-dimensional live imaging of perspiration on the mouse toe was performed by injecting JSAC, a novel dye cocktail of three dyes, into the foot-pad of C57BL/6 mice, followed by an injection of pilocarpine. Live imaging with TPM (Fig. 5a) clearly showed the spiral structure of the acrosyringium in mouse toe (Fig. 5b and Supplementary Movie. 2). Both the stratum-corneum-weighted (Fig. 5c and Supplementary Movie. 3) and the epidermis-weighted (Fig. 5d and Supplementary Movie. 4) images showed that the fluorescein (green) of JSAC entered the sweat ducts (data not shown), while the inner walls of the sweat ducts were stained with sulforhodamine 101 (SR101, red) and epidermal keratinocytes around the acrosyringium with LipiORDER® (light green) (Fig. 5d). After perspiration, the sweat ducts closed, such that the SR101 signal faded (data not shown). These results demonstrated the validity of our three-dimensional live imaging system in examining perspiration in the mouse toe.

### A laser ablation technique to establish an eccrine sweat leakage model in mouse epidermis in turn induces intraepidermal vesicle formation

**Figure 6:**
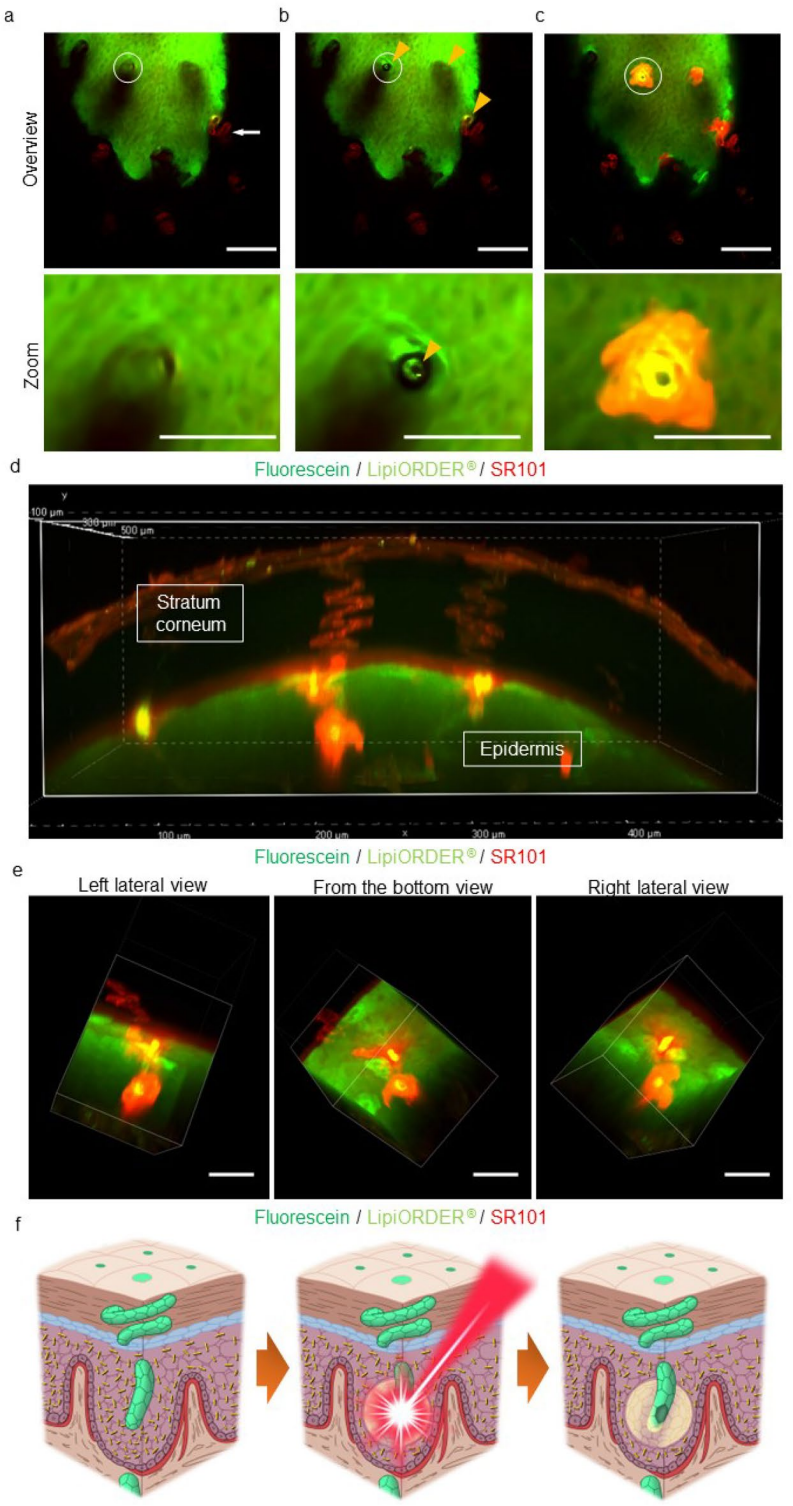
Laser ablation technique induces eccrine sweat leakage and subsequent intraepidermal vesicle formation in the mouse epidermis. (**a**) Z-axis view of a three-dimensional TPM image of the moment of perspiration on a mouse toe stained with a mixture of three dyes: fluorescent solvatochromic pyrene probe (light green), fluorescein (green), and SR101 (red). The white arrow shows the acrosyringium in the stratum corneum. Magnified areas are marked with white circles. Scale bar = 100 µm. (**b**) LASER-snipe. Orange arrowheads indicate the sniped acrosyringium. Magnified areas are marked with white circles. Scale bar = 100 µm. (**c**) Leakage of eccrine sweat from the LASER-sniped acrosyringium. Magnified areas are marked with white circles. Scale bar = 100 µm. (**d**) Three-dimensional view of (**c**). (**e**) An intraepidermal vesicle in (**d**) in left lateral, bottom, and right lateral views. Scale bar = 100 µm. (**f**) Schematic diagram showing the mechanism of intraepidermal vesicle formation.

An eccrine sweat leakage model in mouse epidermis was created by adapting laser ablation for skin tissue^21^. To optimize the laser illumination conditions, the lining cells of the acrosyringium were disrupted, without damage to the surrounding tissue (Fig. 6a,b and Supplementary Movie. 5). This TPM laser ablation technique, named "LASER-snipe”, allowed observation of dye leakage from the acrosyringium using time-lapse TPM (Fig. 6c and Supplementary Movie. 6), confirming our eccrine sweat leakage model in mouse epidermis. A three-dimensional view of the moment of leakage (Fig. 6d and Supplementary Movie. 7) revealed that eccrine sweat leakage in the mouse epidermis occurred in a concentric fashion and mimicked the shape of an intraepidermal vesicle in human PPP (Fig. 6e and Supplementary Movie. 8). We therefore succeeded in inducing formation of an intraepidermal vesicle around the sniped acrosyringium (Fig. 6f).

## Discussion

The sweating function has been the focus of many studies^1^ but advanced morphological studies of eccrine sweat glands have been hindered by the lack of significant progress in the required histopathological methods. In addition to classical H&E staining of tissue sections, immunohistochemistry and in situ hybridization have enabled the visualization of protein and mRNA expression in tissues. However, these techniques require fixed thin sections and are thus only able to reveal expression patterns in dead cells and tissues. In the field of perspiration research using human tissues, there is a need for methods allowing the observation of cell and tissue reactivity as well as morphological changes over time. Ideally, this would be achieved by observing the three-dimensional tissue architecture and physiological or biochemical changes at the tissue and cell level under direct observation.

We developed a method to observe eccrine sweat gland structures based on a three-dimensional, deep-imaging technique using a new pyrene probe and TPM, which allows observation of the three-dimensional structures of eccrine sweat glands ex vivo under direct vision^22^. This technique was successfully applied in a previous study to observe vesicles and pustules in PPP in 3D under direct vision^10^. However, this technique did not allow microscopy observations of physiological sweat flow in real-time, which is necessary to study the pathomechanism of PPP.

In Japan, PPP is a relatively common chronic inflammatory palmoplantar skin disease that is often refractory to treatment^23^. The characteristic histological feature of PPP is intraepidermal vesicle formation preceding pustule development^4,8^. In previous studies, our group demonstrated the presence of eccrine sweat-derived antimicrobial peptides in PPP vesicles^8,9,24^, decreased sweating in the lesional vs. non-lesional area^8^, and partial damage to the structure of the acrosyringium, the source of eccrine sweat in the epidermis, at the site of PPP lesions^10^. These findings strongly suggested a relationship between intraepidermal PPP vesicle formation and eccrine sweat leakage in the epidermis. However, direct evidence that eccrine sweat leaking from the acrosyringium is the cause of intraepidermal vesicle formation was lacking.

Intraepidermal vesicle formation, or epidermal acantholysis, occurs in various skin diseases such as pemphigus, Darier disease, Hailey-Hailey disease, transient acantholytic dermatosis, and miliaria^25–28^. Several studies have shown that the disruption of desmosomes or adherens junctions, essential structures for cell–cell contact^19^, plays a role in intraepidermal vesicle formation, or epidermal acantholysis, in pemphigus, Darier disease, and Hailey-Hailey disease^26–28^. Hence, we initially investigated the expression pattern of intercellular adhesional molecules (for desmosomes: desmoglein-1 and desmoglein-3, for adherens junction: E-cadherin) in the lesional epidermis of PPP patients. Among the studied markers, only a decrease in the membrane expression of E-cadherin by keratinocytes around the pustulo-vesicles or pustules was observed. We therefore hypothesized that decreased E-cadherin expression in the epidermis is the initial step in intraepidermal vesicle formation in PPP.

Human eccrine sweat is mainly composed of water, but also contains electrolytes, bicarbonates, lactic acids, amino acids, heavy metals, immunoglobulins, proteases, protease inhibitors, and cytokines such as IL-1α, IL-1β, IL-6, IL-8, IL-31, and tumor necrosis factor (TNF)-α^1,29^. A previous study showed that IL-1α and IL-1β are highly concentrated (6–30 ng/ml, mostly IL-1α) in human sweat, especially that from the palms and soles^15,16^. In another study, IL-1β (10 ng/ml) stimulation was shown to induce a loss of E-cadherin expression by primary NHEKs and almost complete cell dissociation, whereas the effects of transforming growth factor-β, TNF-α, interferon-γ, and lipopolysaccharide were weak^17^. In the present study, immunocytochemical and flow cytometric analyses showed that both IL-1β and IL-1α lead to a decrease in E-cadherin expression in primary NHEKs. Consistent with the above-cited study, continuous stimulation by IL-1α or IL-1β was shown to reduce E-cadherin expression in the epidermis of the C57BL/6 mouse toe, based on immunohistochemical analysis in which drug delivery was achieved using an osmotic pump system. Taken together, these results implied that IL-1 can disrupt adherens junctions both in vitro and in vivo.

These results supported our hypothesis regarding the pathogenesis of intraepidermal vesicle formation in PPP. Following damage to the acrosyringium, IL-1-rich eccrine sweat leaks into the epidermis, disrupting E-cadherin expression on keratinocytes and in turn leading to intraepidermal vesicle formation. Support for this hypothesis required establishing a link between eccrine sweat leakage and vesicle formation in a living skin equivalent (LSE). Since there is currently no human LSE with eccrine sweat ducts, a mouse model of PPP^30,31^ that also included intraepidermal vesicles on the palmoplantar area of the mouse paw is required. The model described in the present study included those structures and thus allowed us to test our hypothesis of eccrine sweat leakage in the epidermis as the cause of vesicle formation. The model was explored using two techniques with TPM, described in the following.

Three-dimensional, deep imaging with TPM clearly showed that, like the human toe, the mouse toe contains numerous eccrine sweat glands. Using our newly developed fluorescent dye cocktail, JSAC, made up of SR101, fluorescein, and LipiORDER^®^, we were able to follow sweat flow histologically and visualize the structure of the acrosyringium, including its spiral structure, inner walls, and surrounding keratinocytes, in the living mouse foot-pad epidermis. Moreover, JSAC allowed the successful depiction of pilocarpine-induced sweating from eccrine sweat glands in the mouse foot-pad (Fig. 5) and thus, for the first time, live imaging of perspiration in the mouse toe.

We also developed a novel eccrine sweat leakage mouse model using a laser ablation technique, “LASER-snipe,” for use in combination with both JSAC-mediated detection of the lining cells of the acrosyringium in living mouse toe and TPM. In LASER-snipe, one laser pulse kills a few acrosyringium cells. The formation of the equivalent of an intraepidermal vesicle in the intercellular space after leakage of eccrine sweat was confirmed around the LASER-sniped acrosyringium. This finding demonstrated that the leakage of eccrine sweat into the mouse toe-pad epidermis can disrupt intercellular adhesion and induce intraepidermal vesicle formation, thus supporting our hypothesis on the pathogenesis of intraepidermal vesicle formation in PPP. This is also the first study to show live sweat leakage from the acrosyringium by microscopy.

Nonetheless, our study had two limitations. First, the exact mechanism of eccrine sweat leakage from the acrosyringium remains unclear; while it likely involves the disruption of tight junctions in the lining cells, the responsible factors are so far unknown. A study of lesional skin in atopic dermatitis reported the decreased expression of claudin-3, a tight junction component, in sweat glands, accompanied by sweat leakage in the dermis^32^. It also showed reduced claudin expression in the histamine-treated dermal sweat duct of the mouse footpad, resulting in tracer leakage into the paracellular space^32^. Although the presence of one of the tight junction-associated proteins occludin in the acrosyringium of human skin has also been described^33^, the barrier system for sweat leakage in the acrosyringium has not been clarified. Second, the induction of pustule formation requires neutrophil recruitment into an intraepidermal vesicle, which was not reproduced in our mouse model. The release of IL-8 and IL-36, key cytokines that mediate neutrophil recruitment, in human tonsil epithelial cells and primary NHEKs exposed to cigarette smoke was also shown to contribute to the pathogenesis of PPP^34^, as was dysbiosis of the oral microbiota^35–37^. In a previous study, our group detected a microbiome in sterile pustules of PPP^38^. From these perspectives, our mouse model would benefit from extrinsic stimulation, such as cigarette smoke and bacterial components in the oral cavity, upper airway, and palms and soles, to recruit neutrophils into the intraepidermal vesicle.

In conclusion, this study sheds light on the pathogenesis of PPP. According to our model, mechanical or functional damage to the acrosyringium in the palms and soles by a yet unidentified stimulant causes IL-1-rich eccrine sweat to leak into the epidermis. IL-1 disrupts E-cadherin expression on keratinocytes, leading to intraepidermal vesicle formation. While this sequence of events was demonstrated in a mouse model of vesicle formation in PPP, the model must be expanded if it is to completely mimic human PPP.

## Methods

### Human skin samples

Three 2-mm punch biopsy specimens were obtained at Ehime University Hospital from three patients diagnosed with PPP based on clinical features, disease course, and the diagnostic histopathological features of the vesicles, i.e., without spongiosis and microabscess on their edges^12^.

### Histological and immunohistochemical analyses of human skin samples

Formalin-fixed, paraffin-embedded blocks of pustulo-vesicles or pustules from biopsy specimens of the three PPP patients were prepared for H&E staining using routine histological techniques. For immunohistochemical analysis of intercellular adhesional molecules in the lesional epidermis, 5-µm tissue sections were deparaffinized, hydrated, and subjected to antigen retrieval by heat mediation either in citrate buffer, pH 6, or in antigen retrieval solution (415211; Nichirei, Tokyo, Japan), pH 9, according to the manufacturer's protocol. The tissue sections were then incubated overnight with primary antibodies [anti-E-cadherin (1:500; Abcam, ab40772), anti-desmoglein-1 (1:500; Abcam, ab209490), and anti-desmoglein-3 (1:250; Abcam, ab183743)] in background-reducing antibody diluent (Agilent, S3022) in a humidified chamber at 4 °C. An Alexa-Fluor 488-conjugated goat anti-rabbit IgG (1:200) was used as the secondary antibody. Nuclei were counterstained with propidium iodide. The stained tissue sections were examined by light and fluorescence microscopy (BZ-X800, Keyence Corp., IL, USA).

### Cell culture

Primary NHEKs isolated from surgically discarded neonatal skin samples were cultured in MCDB153 medium supplemented with the appropriate reagents as previously described^29^. Cells used for the experiments were in their fourth passage, and subconfluent cultures were subjected to stimulation.

### Immunocytochemical assay

Primary NHEKs were seeded and cultured in EpiLife™ medium containing 0.06 mM calcium (Gibco) and supplemented with 5 ml of Epilife™ defined growth supplement (EDGS, Gibco) in 8-well chamber slides coated with type I collagen. After 48 h, the medium was replaced with EpiLife™ medium containing 1.2 mM calcium to facilitate cell differentiation. After an additional 48 h, cells were stimulated for 18 h with 10, 100, or 1000 ng IL-1α or IL-1β/ml^17^ and processed for immunocytochemical staining. For the assay, cells on slides were fixed with 4% paraformaldehyde in PBS for 30 min at room temperature and then incubated overnight with an anti-E-cadherin rabbit monoclonal primary antibody (1:500; Abcam, ab40772) in background-reducing antibody diluent (Agilent, S3022) in a humidified chamber at 4 °C. An Alexa-Fluor 488-conjugated goat anti-rabbit IgG (1:200) served as the secondary antibody. Finally, the nuclei were counterstained with Hoechst dye. The stained tissue sections were examined by light and fluorescence microscopy (BZ-X800, Keyence Corp., IL, USA).

### Flow cytometric analysis

Primary NHEKs were seeded and cultured in EpiLife™ medium containing 0.06 mM calcium (Gibco) and supplemented with 5 ml of EDGS (Gibco) on 60-mm type-I-collagen-coated dishes. After 48 h, the medium was replaced with EpiLife™ medium containing 1.2 mM calcium without EDGS to facilitate cell differentiation. After 48 h, the cells were stimulated with 10 ng of IL-1α or IL-1β/ml for 18 h and prepared for flow cytometry. Specifically, the cells were trypsinized, adjusted to a concentration of 1 × 10^6^ cells per 100 μl PBS, incubated with 10 μl of phycoerythrin-conjugated anti-E-cadherin mouse primary monoclonal antibody (Abcam, ab128125) for 30 min at room temperature, washed, and analyzed for E-cadherin expression using multicolor flow cytometry (Gallios, Beckman Coulter). The data were analyzed using FlowJo™ v7.6.5 software (Becton, Dickinson and Company, New Jersey, USA).

### Mice

Six- to 22-week-old C57BL/6 mice originally purchased from CLEA Japan (Tokyo, Japan) were used in this study. The mice were housed in the animal facility at Ehime University in a specific pathogen-free environment at 23 ± 2 °C with a 12 h/12 h dark/light cycle. All mice were anesthetized with mixture of medetomidine(0.3 mg/kg), midazolam(4.0 mg/kg), and butorphanol(5.0 mg/kg) by intraperitoneal injection and euthanized with carbon dioxide by oral inhalation.

### IL-1 treatment and E-cadherin expression analysis of mouse skin

For the continuous intraepidermal stimulation assays, C57BL/6 mice were implanted with an Alzet^®^ osmotic pump (Muromachi Kikai Co., Ltd., Tokyo, Japan) and cannula under general anesthesia for continuous intraepidermal administration of 10 ng IL-1α or IL-1β/ml (3 µl/h; two animals, respectively) or PBS (two animals for positive control) to the toes for 24 h (Fig. 4a–c). Non-treated toes served as an additional positive control. After stimulation, the mice were euthanized under general anesthesia. The toes were isolated using scissors and fixed with 4% paraformaldehyde in PBS for 2 h at room temperature. After dehydration, the samples were paraffin-embedded and 5-µm tissue sections were prepared for immunohistochemical staining. The tissue sections were deparaffinized, hydrated, and subjected to antigen retrieval by heat mediation in a citrate buffer, pH 6, according to the manufacturer's protocol followed by incubation overnight with an anti-E-cadherin rabbit monoclonal primary antibody (1:80,000; Abcam, ab76319) in background-reducing antibody diluent (Agilent, S3022) in a humidified chamber at 4 °C. An Alexa-Fluor 488-conjugated goat anti-rabbit IgG (1:200) was used as the secondary antibody. Cell nuclei were counterstained with Hoechst dye. The stained tissue sections were examined by confocal laser scanning microscopy using a Nikon A1R MP + multiphoton confocal microscope at 403 and 488 nm wavelength excitation. Subsequently, the sections were processed for H&E staining using routine histological techniques.

### Three-dimensional histopathological analysis of mice eccrine glands

C57BL/6 mice were euthanized after general anesthesia, and the paw was removed for three-dimensional imaging of the eccrine sweat glands in the toe. The samples were fixed overnight in 4% paraformaldehyde in PBS at 4 °C, optically cleared, and stained using the LUCID method and a pyrene probe (LipiORDER^®^; Funakoshi Co., Ltd., Tokyo, Japan), according to a previously described protocol^19^.

### JSAC, a novel fluorescent cocktail for the live imaging of sweat from mice eccrine glands

For the three-dimensional live imaging of perspiration by mouse toe, C57BL/6 mice were placed under general anesthesia and retro-orbitally injected with our JSAC dye mixture, consisting of SR101 (Sigma, 5 mg/ml), fluorescein (Sigma, 5 mg/ml), and LipiORDER^®^ (100 μM) in 100 μl PBS. The foot-pad of each mouse was subcutaneously injected with 50 μg of pilocarpine (Wako Junyaku Co., Ltd., Osaka, Japan) in 20 μl PBS to induce perspiration. Subsequently, the toe-pad was observed by time-lapse TPM, as described below.

### Two-photon excitation microscopy

A Nikon A1R MP + multiphoton confocal microscope (MaiTai eHP DeepSee, Spectra-Physics) with an Apo LWD 25 × 1.10W objective lens was used for TPM. All 3D images were acquired at 1024 × 1024 (0.51 mm/pixel) with 0.5-fps and 2.0-µm Z steps. Fluorescein signals were detected at excitation (ex)/emission (em) wavelengths of 880/500–550 nm, SR101 fluorescence signals at ex/em wavelengths of 880/593–750 nm, and LipiORDER^®^ signals at ex/em wavelengths of 880/500–550 nm, 560–593 nm, and 593–750 nm.

### LASER-snipe: a technique for inducing eccrine sweat leakage in mouse epidermis

C57BL/6 mice were placed under general anesthesia and the JSAC dye mixture was retro-orbitally injected into one. Laser ablation of the detected lining cell of the acrosyringium in the mouse toe using our LASER-snipe technique was then performed under TPM. A laser pulse of 880 nm was delivered for ~ 8 s with 100% laser power into a 2-µm^2^ area of the toe. Ablation was followed by subcutaneous injection of 50 μg of pilocarpine in 20 μl PBS into the toe-pad of each mouse to induce perspiration. Subsequently, the toe-pad was observed by time-lapse TPM.

### Study approval

Human participant studies were approved by the Institutional Ethics Committee of Ehime University School of Medicine (approval number: 18022009) and were performed in accordance with the principles of the Declaration of Helsinki. Informed consent to collect and evaluate skin biopsy specimens was obtained from all patients. All animal experiments were approved by the Institutional Animal Care and Use Committee of Ehime University (approval number: 05NE49-1,16) and conformed with the NIH guidelines for the care and use of animals, the recommendations of the International Association for the Study of Pain, and the ARRIVE guidelines. All efforts were made to minimize suffering.

### Supplementary Information

Supplementary Information.

Supplementary Video 1.

Supplementary Video 2.

Supplementary Video 3.

Supplementary Video 4.

Supplementary Video 5.

Supplementary Video 6.

Supplementary Video 7.

Supplementary Video 8.

## Data Availability

The datasets generated during and/or analysed during the current study are available from the corresponding author on reasonable request.
