# Supplementary material for: A fluorescence imaging technique suggests that sweat leakage in the epidermis contributes to the pathomechanism of palmoplantar pustulosis
Source: Sci Rep. 2024 Jan 3;14:378. doi: 10.1038/s41598-023-50875-x (PMC10764317; doi:10.1038/s41598-023-50875-x)
Supplement: Supplementary file 1 — Supplementary Information. [file 41598_2023_50875_MOESM1_ESM.docx]

**A fluorescence imaging technique suggests that sweat leakage in the epidermis contributes to the pathomechanism of palmoplantar pustulosis**

Kazuki Yatsuzuka^1^, Ryosuke Kawakami^2^, Yosuke Niko^3^, Teruko Tsuda^1^, Kenji Kameda^1^, Nobushige Kohri^1^, Satoshi Yoshida^1^, Ken Shiraishi^1^, Jun Muto^1^, Hideki Mori^1^, Yasuhiro Fujisawa^1^, Takeshi Imamura^2^, Masamoto Murakami^1^*

1 Department of Dermatology, Ehime University Graduate School of Medicine, Ehime, Japan

2 Department of Molecular Medicine for Pathogenesis, Ehime University Graduate School of Medicine, Ehime, Japan

3 Research and Education Faculty, Multidisciplinary Science Cluster, Interdisciplinary Science Unit, Kochi University, Kochi, Japan

Corresponding author*:

Masamoto Murakami

Department of Dermatology, Ehime University Graduate School of Medicine, Toon, Ehime 791-0295, Japan

Phone: +81 89-960-5350

E-Mail: mamuraka@m.ehime-u.ac.jp

**
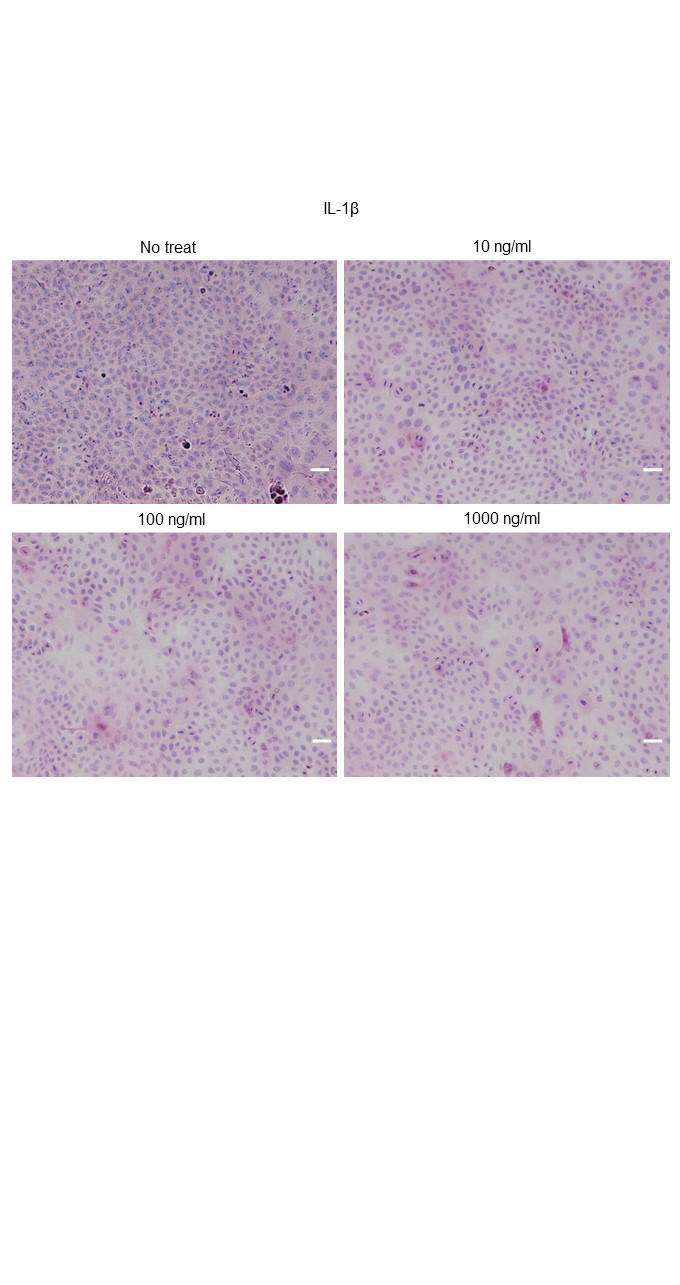
Supplementary Fig. 1 Morphological comparison of primary NHEKs stimulated with IL-1β**

H&E staining of each specimen in Fig. 2 (b, c). Scale bar = 100 µm.

**Supplementary Movie. 1 Three-dimensional, deep-imaging with TPM shows numerous eccrine sweat glands in the mouse toe pad**

Three-dimensional movie of Fig. 3 (a). Scale bar = 100 µm.

**Supplementary Movie. 2 Three-dimensional, deep-imaging with TPM using a novel dye mixture enables live imaging of perspiration from the mouse toe-pad**

Three-dimensional movie of Fig. 5 (b).

**Supplementary Movie. 3 Three-dimensional, deep-imaging with TPM using a novel dye mixture enables live imaging of perspiration from the mouse toe-pad**

Three-dimensional movie of Fig. 5 (c).

**Supplementary Movie. 4 Three-dimensional, deep-imaging with TPM using a novel dye mixture enables live imaging of perspiration from the mouse toe-pad**

Three-dimensional movie of Fig. 5 (d).

**Supplementary Movie. 5 LASER-snipe**

Time-lapse movie of Fig. 6 (a, b). Scale bar = 100 µm.

**Supplementary Movie. 6 Leakage of eccrine sweat from the LASER-sniped acrosyringium**

Time-lapse (80 min) movie of Fig. 6 (c). Scale bar = 100 µm.

**Supplementary Movie. 7 LASER-snipe induces eccrine sweat leakage and subsequent intraepidermal vesicle formation in the mouse epidermis**

Three-dimensional movie of Fig. 6 (d).

**Supplementary Movie. 8 LASER-snipe induces eccrine sweat leakage and subsequent intraepidermal vesicle formation in the mouse epidermis**

Three-dimensional movie of Fig. 6 (e).

The English in this document has been checked by at least two professional editors, both native speakers of English. For a certificate, please see:

<http://www.textcheck.com/certificate/gMz8bM>
