# Supplementary figures and images for: A fluorescence imaging technique suggests that sweat leakage in the epidermis contributes to the pathomechanism of palmoplantar pustulosis
Source: Sci Rep. 2024 Jan 3;14:378. doi: 10.1038/s41598-023-50875-x (PMC10764317; doi:10.1038/s41598-023-50875-x)

## Slide 1
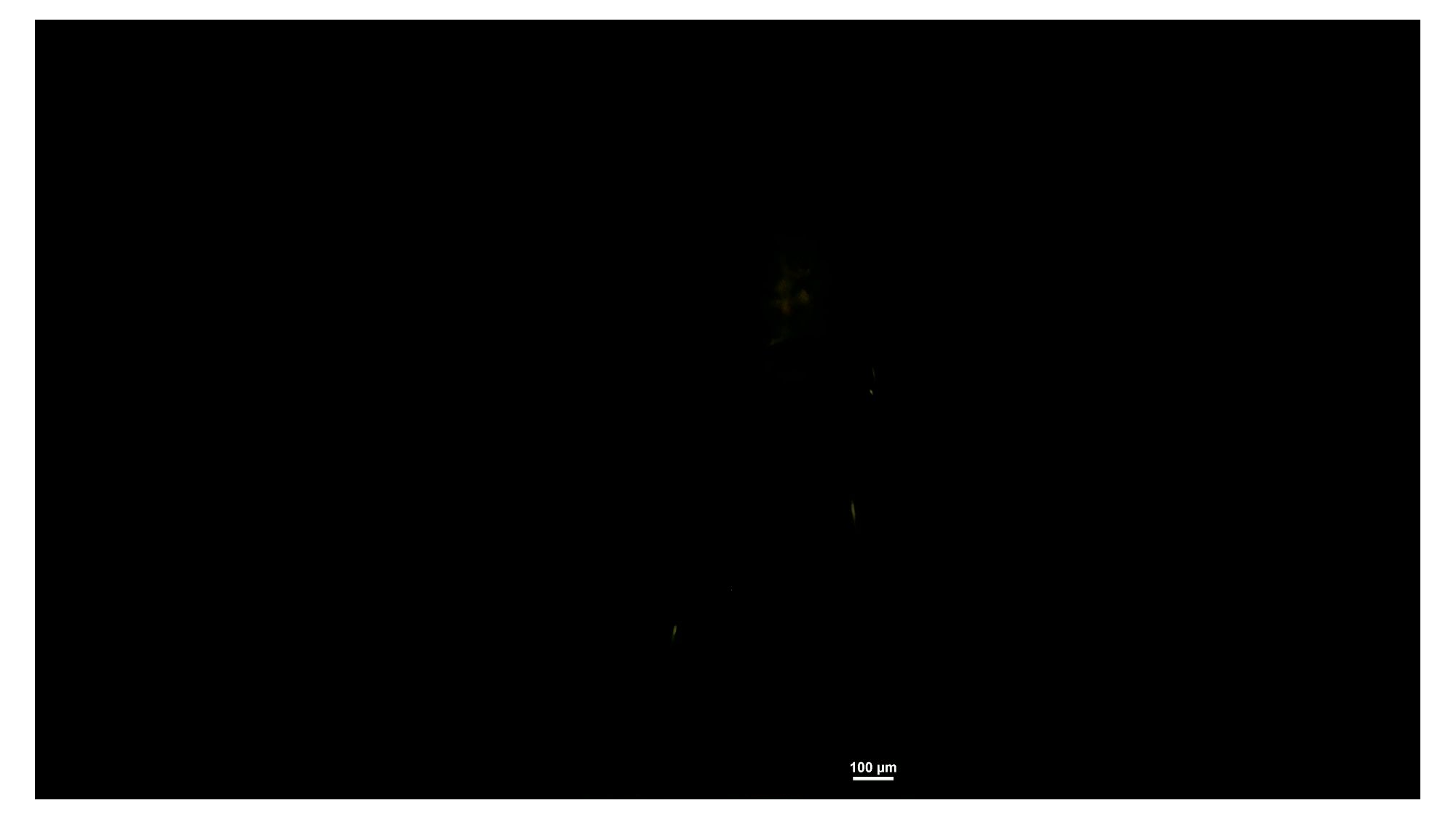

Supplement: Supplementary file 2 — Supplementary Video 1. [file 41598_2023_50875_MOESM2_ESM.pptx]

## Slide 1
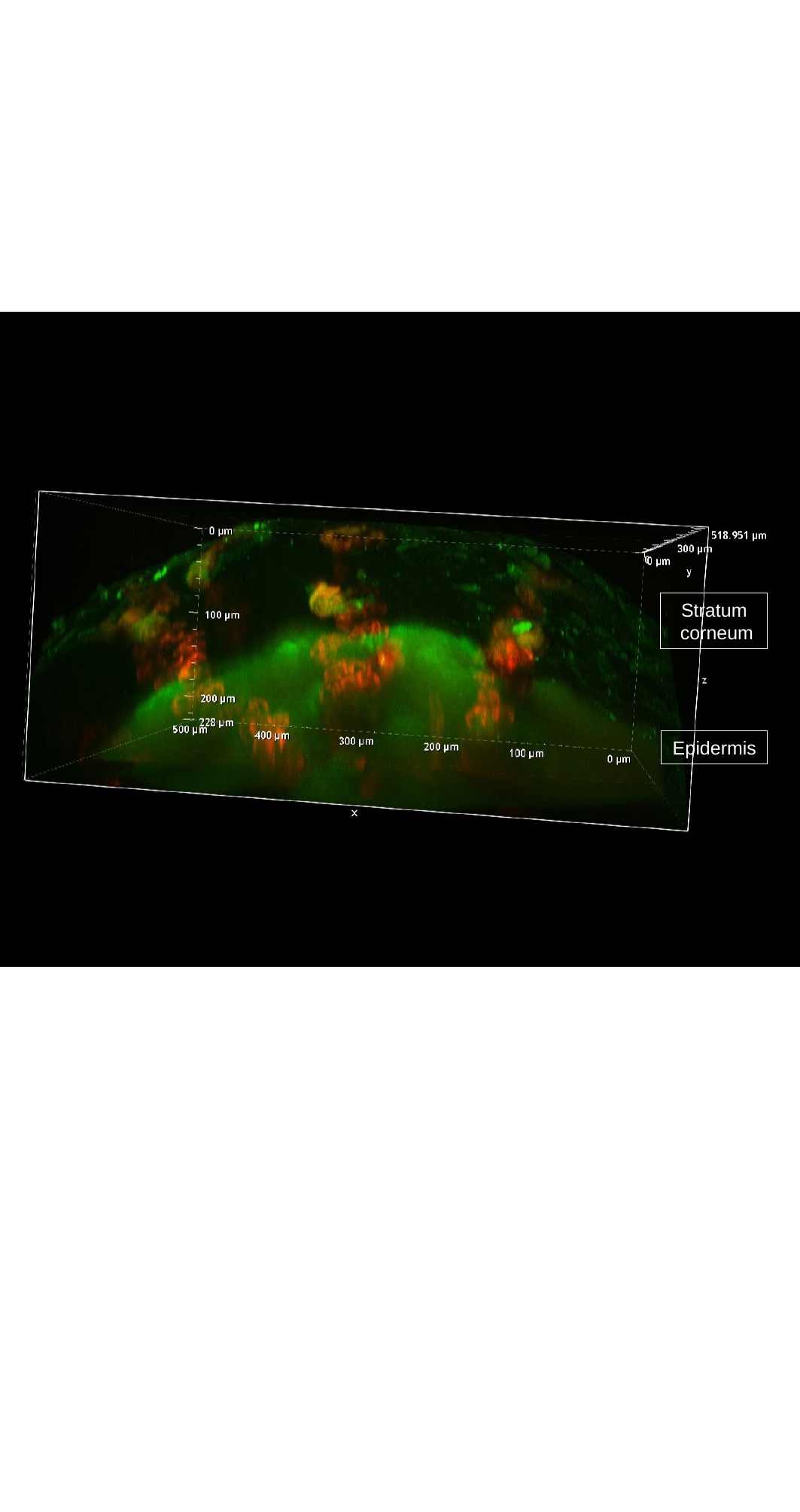

Stratum
 corneum
Epidermis

Supplement: Supplementary file 3 — Supplementary Video 2. [file 41598_2023_50875_MOESM3_ESM.pptx]

## Slide 1
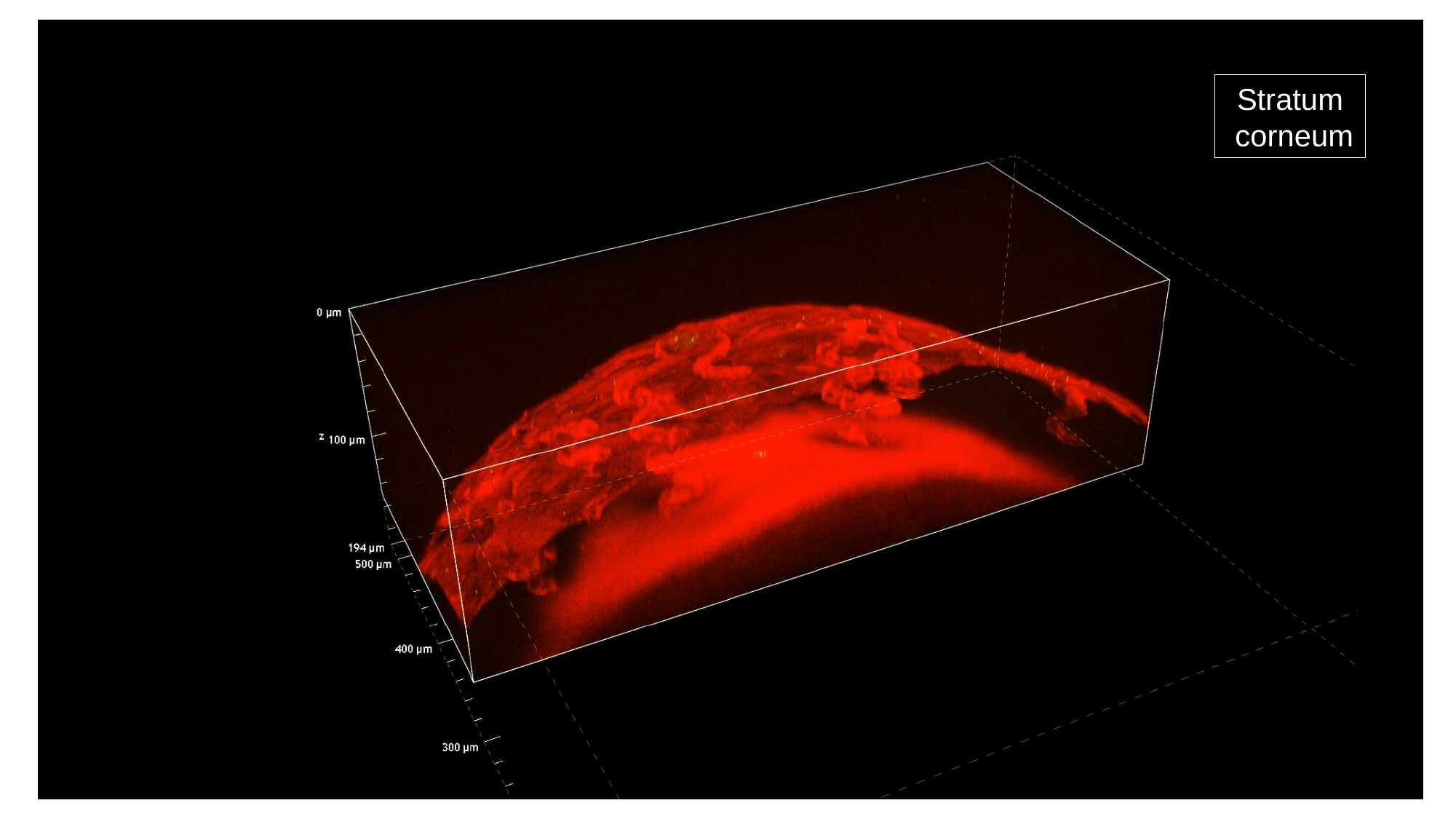

Stratum
 corneum

Supplement: Supplementary file 4 — Supplementary Video 3. [file 41598_2023_50875_MOESM4_ESM.pptx]

## Slide 1
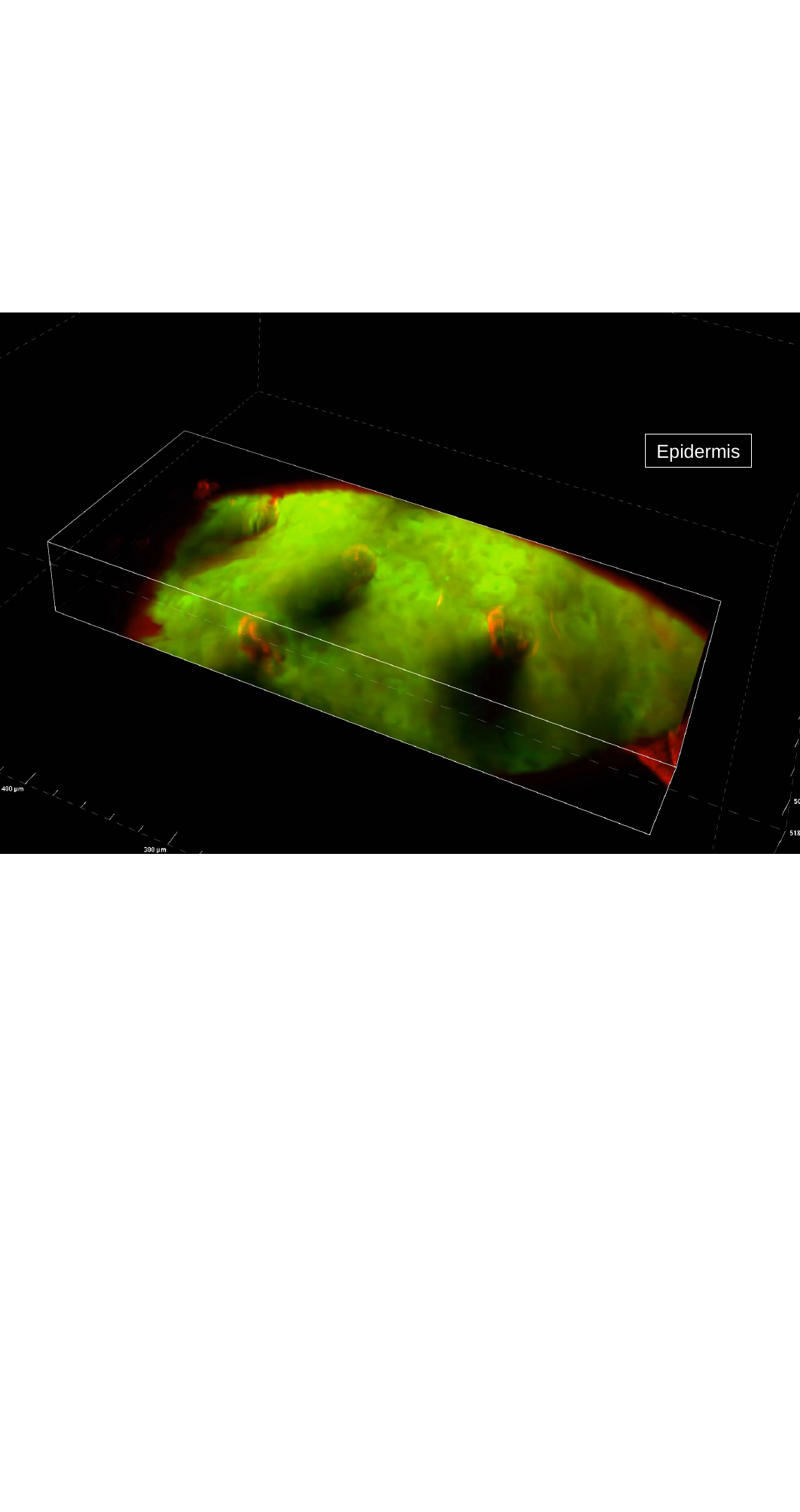

Epidermis
Stratum
 corneum
Epidermis

Supplement: Supplementary file 5 — Supplementary Video 4. [file 41598_2023_50875_MOESM5_ESM.pptx]

## Slide 1
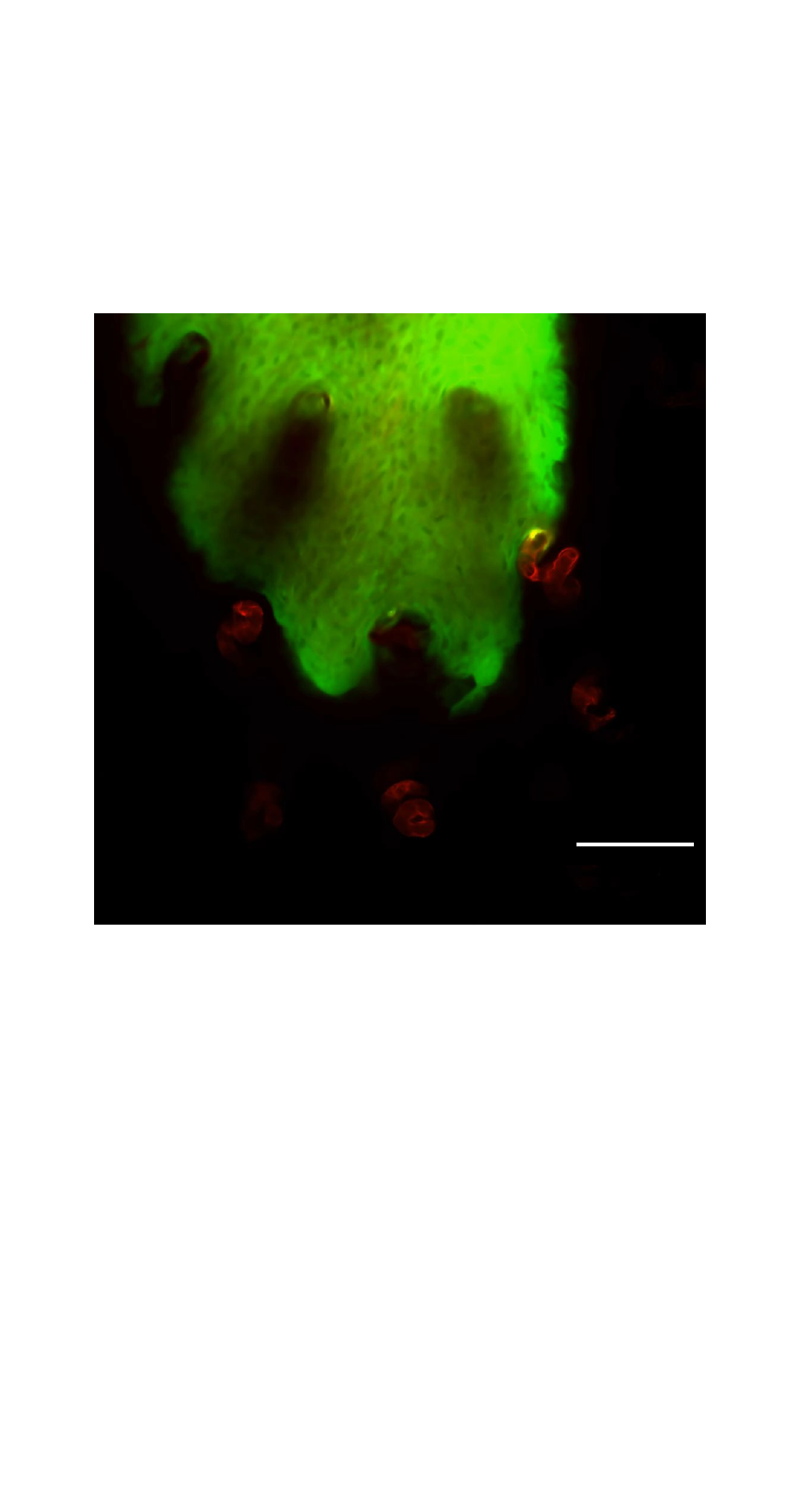

Supplement: Supplementary file 6 — Supplementary Video 5. [file 41598_2023_50875_MOESM6_ESM.pptx]

## Slide 1
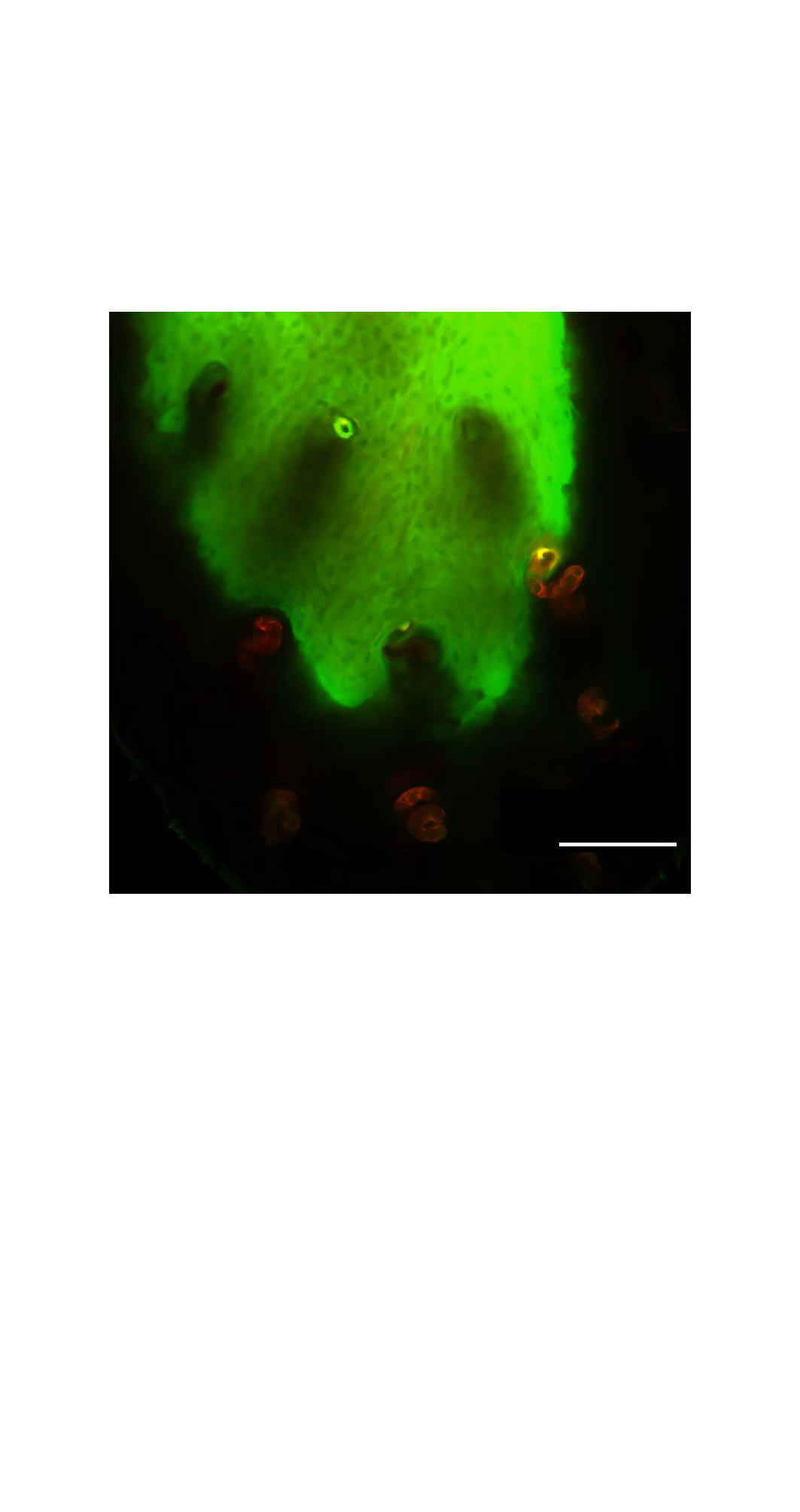

Supplement: Supplementary file 7 — Supplementary Video 6. [file 41598_2023_50875_MOESM7_ESM.pptx]

## Slide 1
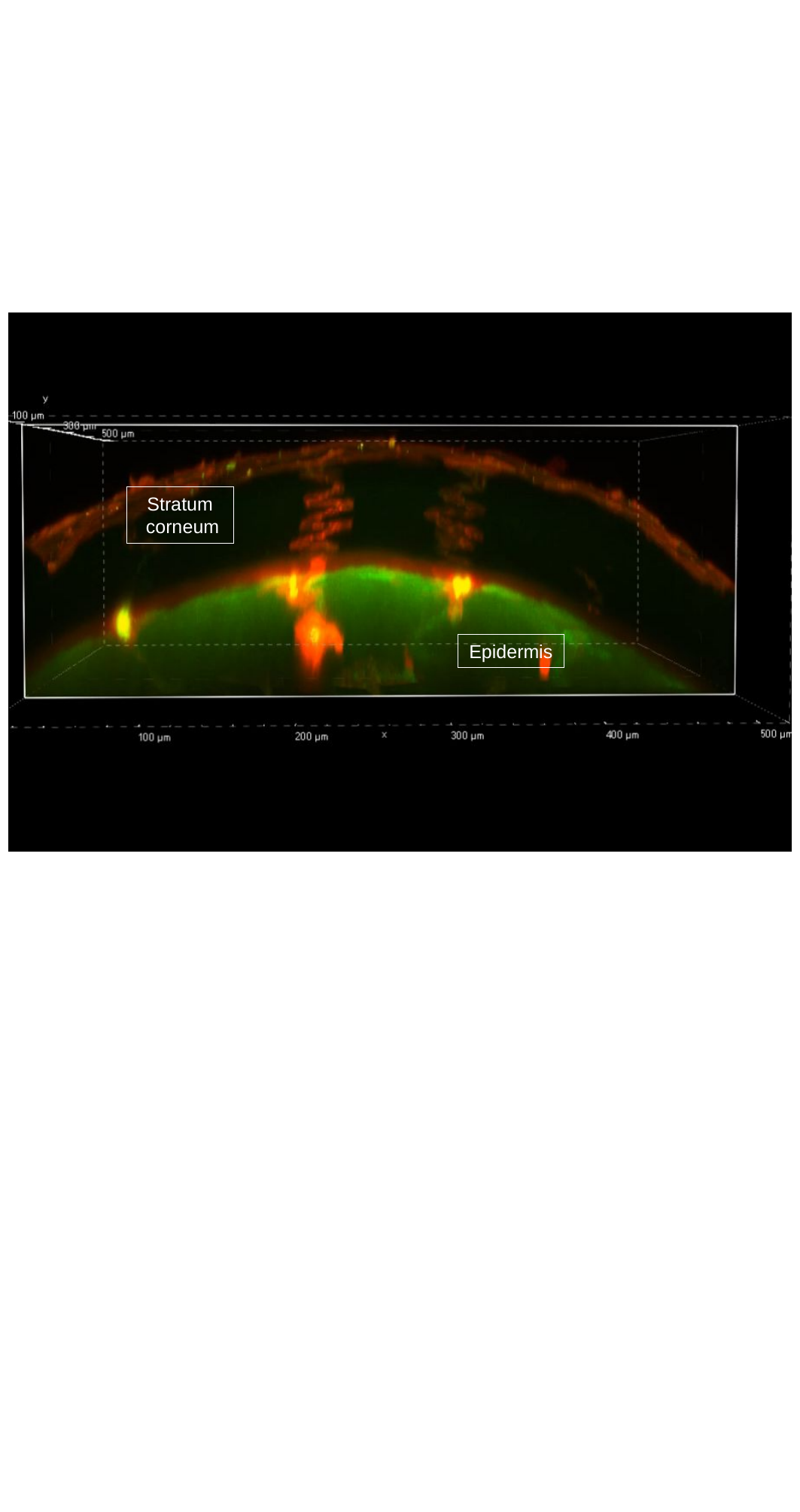

Stratum
 corneum
Epidermis

Supplement: Supplementary file 8 — Supplementary Video 7. [file 41598_2023_50875_MOESM8_ESM.pptx]

## Slide 1
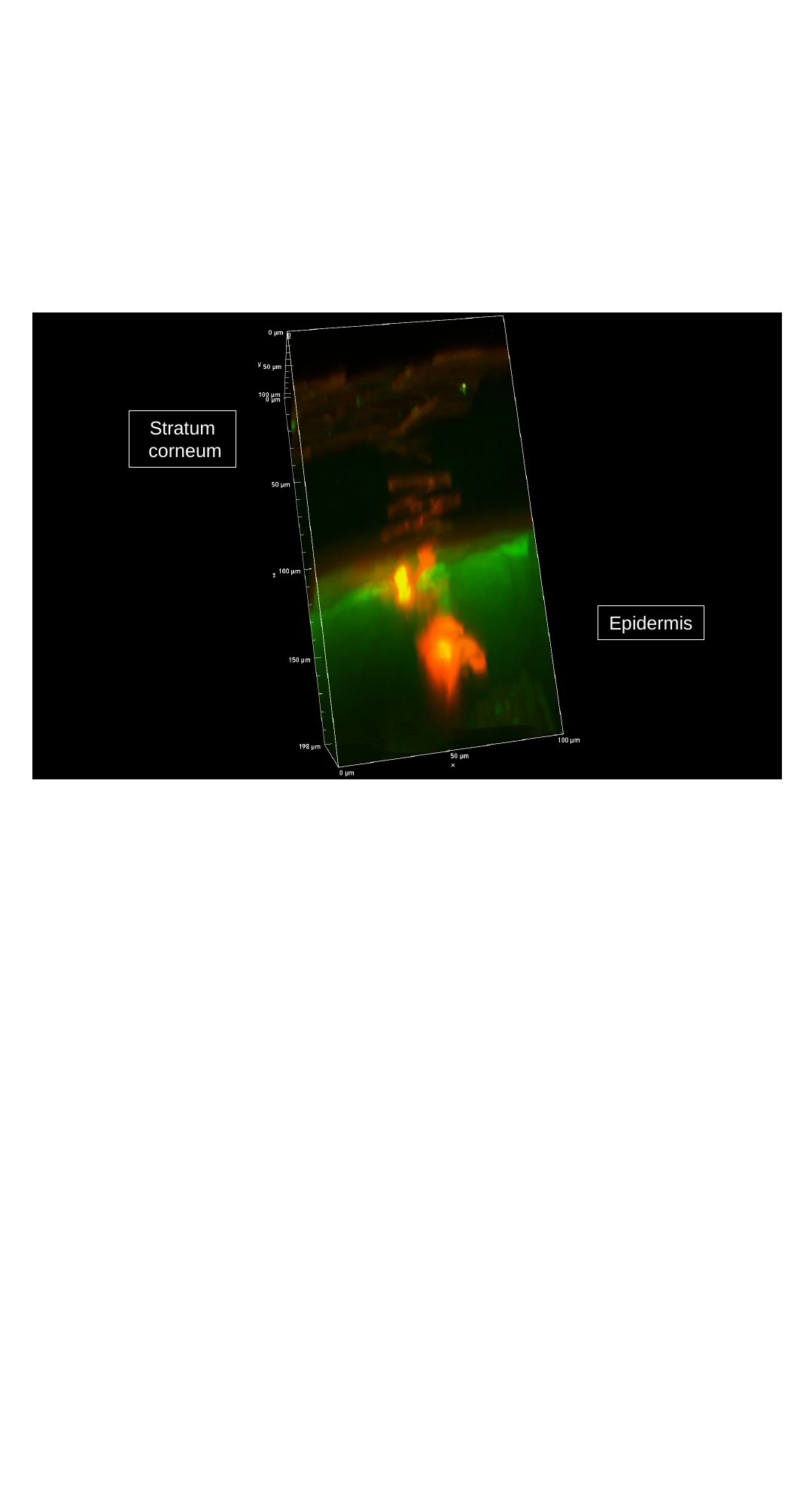

Stratum
 corneum
Epidermis
Stratum
 corneum
Epidermis

Supplement: Supplementary file 9 — Supplementary Video 8. [file 41598_2023_50875_MOESM9_ESM.pptx]
